# Nuclear Architecture Related 1-Dependent H_2_ Reshapes Root Plasticity Through Targeting Root Meristem Cell Division Activity

**DOI:** 10.3390/antiox15101228

**Published:** 2026-09-23

**Authors:** Ke Jiang, Pengfei Cheng, Ziyu Liu, Hongmei Du, Wenbiao Shen

**Affiliations:** 1 Laboratory Center of Life Sciences, College of Life Sciences, Nanjing Agricultural University, Nanjing 210095, China; 2022216005@stu.njau.edu.cn (K.J.); t2024051@njau.edu.cn (P.C.); 2025216044@stu.njau.edu.cn (Z.L.); 2 School of Design, Shanghai Jiao Tong University, Shanghai 200240, China; hmdu@sjtu.edu.cn

**Keywords:** molecular hydrogen, cell division, auxin signaling, ROS homeostasis

## Abstract

Root system architecture plasticity is critical for optimizing anchorage and nutrient and water uptake. However, whether and how biogenic molecular hydrogen (H_2_) influences root plasticity remains further explored. Here, using ABA-inhibited plant root growth as a research model, we show that *Arabidopsis* hydrogenase Nuclear Architecture Related 1 (NAR1)-dependent H_2_ reshapes root plasticity. First, we observed that *NAR1* overexpression promotes primary root elongation and meristem cell division, whereas *RNAi-NAR1* lines show impaired root growth, particularly upon ABA administration. Consistently, ABA-inhibited *CYCB1;1* expression, a G2/M marker, was substantially recovered or aggravated in *OE-NAR1* or *RNAi-NAR1* lines. Further analysis revealed that NAR1 preserves DR5 activity, and AUX1, PIN1, and PIN2 signals under ABA. In parallel, NAR1 sustains cellular homeostasis by limiting ABA-triggered oxidative stress, as evidenced by upregulation of antioxidant defense and reduced ROS and lipid peroxidation. The functional requirement for NAR1-derived H_2_ was further corroborated by H_2_ supplementation, which effectively rescued the inhibited phenotypes of *RNAi-NAR1* lines. Together, our findings reveal a convergence of meristem cell division activity, auxin signaling and ROS homeostasis governed by NAR1-dependent H_2_, which reshapes root plasticity under ABA treatment. These observations provide new molecular insights into endogenous H_2_ signaling for shaping plant plasticity in complex environments.

## 1. Introduction

Root system architecture (RSA) serves as the primary interface for nutrient and water uptake from the soil, with its developmental plasticity being indispensable for plant adaptation to fluctuating environments [1]. The developmental plasticity of the primary root is largely driven by the activity of the root apical meristem (RAM), where a precise equilibrium between cell proliferation and differentiation must be efficiently maintained to ensure sustained growth [2]. This complex process is orchestrated by sophisticated signaling networks where various phytohormones integrate environmental cues into endogenous growth programs.

As a key stress-responsive hormone, abscisic acid (ABA) is known to exert a potent inhibitory effect on primary root elongation, particularly under adverse conditions [3,4,5]. This inhibition is partly ascribed to the impairment of auxin homeostasis and cell-cycle progression [6], as evidenced by the downregulation of polar auxin transporters [7,8], and the repression of *CYCB1;1*, one of G2/M regulators [9,10]. In addition, ABA-induced growth quiescence is frequently associated with the overaccumulation of reactive oxygen species (ROS), which impairs cellular homeostasis in the RAM [7]. Despite the extensive documentation of these repressive pathways, the endogenous mechanisms that antagonize such inhibition to maintain root growth and architecture remain to be fully elucidated.

Molecular hydrogen (H_2_), historically considered as a metabolic byproduct or a component of the primitive atmosphere, has recently emerged as a potential versatile gaseous signaling molecule in plants [11,12]. It has been reported that H_2_ participates in a wide array of developmental processes, including seed germination, seedling establishment, and various stress responses [13,14,15,16]. Much of the current evidence, however, comes from studies using exogenous H_2_ donors or heterologous expression of algal hydrogenase (*CrHYD1*). Such reliance on non-native systems limited the understanding of the spatiotemporal dynamics and intrinsic regulatory functions of endogenous H_2_ in plants.

The recent identification of Nuclear Architecture Related 1 (NAR1) as a pivotal enzyme responsible for endogenous H_2_ production in *Arabidopsis* [17] and tomato [18] provides a crucial genetic scaffold to further investigate the biological significance of H_2_, since NAR1-catalyzed H_2_ has been shown to facilitate floral transition and promote lateral root branching. Given that H_2_ is known to interact with the auxin-signaling network [15,16], the next question is whether or how the NAR1-H_2_ module may function as an endogenous checkpoint to counteract ABA-induced growth restriction.

By combining genetic and pharmacological approaches, we discovered that NAR1-dependent H_2_ is required for sustaining primary root elongation and meristematic activity, thereby relieving ABA-mediated suppression. Further mechanism analysis clearly revealed NAR1-dependent H_2_ activates auxin signaling and cell-cycle gene expression with concomitant alleviation of oxidative stress. These findings provide new insights into the physiological role of endogenous H_2_ in regulating root plasticity, and offer a theoretical basis for the genetic improvement of RSA in challenging environments.

## 2. Materials and Methods

### 2.1. Plant Materials and Growth Conditions

*Arabidopsis thaliana* (ecotype *Columbia-0*) was used in this study. Plant materials included wild-type (WT), *NAR1* (AT4G16440) RNA interference (RNAi) lines (*RNAi-NAR1-1* and *RNAi-NAR1-2*), and *NAR1* overexpression (*OE*) lines driven by the 35S promoter (*OE-NAR1-1* and *OE-NAR1-2*) [17]. In addition, the auxin-related reporter lines used in this study, including *CYCB1_pro_:GUS*, *DR5rev:3xVENUS-N7*, *PIN1_pro_:PIN1-GFP*, *PIN2_pro_:PIN2-GFP*, and *AUX1_pro_:AUX1-YFP*, were provided by Prof. Wei Xuan (Nanjing Agricultural University) [19]. These reporter lines were subsequently crossed into the *RNAi-NAR1* or *OE-NAR1* genetic background to generate the required hybrids. The hybrid progenies were obtained by hygromycin selection and verified by PCR with specific primers (Table S1).

*Arabidopsis* seeds were surface sterilized with NaClO (5%, *v*/*v*), and then washed three times with sterile water. Seeds were vernalized in darkness at 4 °C for 48 h and then germinated on half-strength Murashige and Skoog (½MS) solid medium containing 1% sucrose and 1.5% agar. All seedlings were grown in a growth chamber set to a 16/8 h light/dark cycle at 23/21 °C, with a light intensity of 180 μmol m^−2^ s^−1^.

To determine the appropriate ABA concentration for subsequent experiments, seedlings were treated with 0, 0.1, 1, 5, and 10 µM ABA for 7 days (Figure S1). Based on these results, 5 µM ABA was selected because it caused significant inhibition of primary root elongation without excessive suppression. The H_2_ concentration (0.5%, *v*/*v*) was selected based on a dose–response assay, in which *RNAi*-*NAR1* seedlings treated with 5 µM ABA were fumigated with 0, 0.01%, 0.1%, 0.5%, or 1% H_2_ (*v*/*v*; Figure S2). For phenotypic analysis, three-day-old seedlings were transferred to ½MS solid medium supplemented with ABA (5 μM) or exposed to exogenous H_2_ fumigation (0.5%, *v*/*v*), with untreated seedlings as the control. Primary root elongation during this period was quantified [20]. Seedling fresh weight was determined as the weight per 50 plants.

For seedling growth and meristem parameters, measurements were performed after 7 days of ABA treatment to assess long-term effects on growth and development. For molecular and physiological assays, assessments were conducted after 3 days of ABA treatment to monitor early responses.

### 2.2. Determination of H_2_ Concentration

H_2_ concentration was quantified by gas chromatography as described previously [15]. Briefly, about 500 mg tissue was homogenized in 2 mL water, acidified with 200 μL 2 M sulfuric acid, and incubated at 70 °C for 20 min prior to headspace GC analysis (GC 7980, Techcomp, Shanghai, China).

### 2.3. Staining and Microscopy

An LSM 800 microscope (Carl Zeiss, Oberkochen, Germany) was used for fluorescence imaging. For morphological analysis, seedlings were stained with FM4-64 at 2 μM for 5 min to visualize the length and cell numbers of root meristematic zone [19]. The quiescent center (QC) was identified directly by morphology when clearly visible, or otherwise by its position at the junction between the columella stem cells and the vasculature initials, following the root apical meristem organization in *Arabidopsis* [2]. The meristematic zone was defined as the region from the QC to the first rectangular cell in the cortex. According to previously described methods [21], cell production rate was calculated by dividing the root elongation rate by the average mature cortical cell length. Root elongation rate was calculated as the final root length divided by the growth duration. Mature cortical cell length was measured at 5 mm from the root tip. The length of the root cap was measured from the QC to the last intact layer (separating layer was not included) [22]. Additionally, the fluorescence signal intensities of VENUS, YFP, and GFP were detected [19], and all values were normalized to those of the control group.

To detect ROS accumulation, seedlings were stained with 10 μM 2′,7′-dichlorodihydrofluorescein diacetate (H_2_DCFDA) for 10 min in the dark, rinsed three times, and observed under an LSM 800 microscope (excitation 488 nm, emission 500–530 nm) [23]. Fluorescence images were captured by ZEN 3.8.1 software for intensity quantification.

### 2.4. 3,3′-Diaminobenzidine (DAB) and Nitro Blue Tetrazolium (NBT) Staining

Histochemical staining was performed to detect hydrogen peroxide (H_2_O_2_) using 0.1% DAB following a previously described method with minor modifications [24]. Seedlings were vacuum-infiltrated and incubated 10 h in the dark, then washed twice with distilled water and examined under a microscope (Axio Observer 3, Carl Zeiss).

Superoxide anion (O_2_^−^) was visualized by NBT staining as described with minor adaptations [24]. Seedlings were immersed in 0.1% NBT, and reacted for 1 h at room temperature in the dark, followed by rinsing with distilled water and microscopic imaging (Axio Observer 3, Carl Zeiss).

### 2.5. Measurement of Thiobarbituric Acid Reactive Substances (TBARS) Content

TBARS content was measured according to a previously reported method with minor adjustments [25]. In brief, about 0.2 g of samples were homogenized with 2 mL trichloroacetic acid (0.1%; *w*/*v*). The homogenate was centrifuged at 12,000× *g* for 15 min, after which 0.5 mL of the supernatant was transferred to a fresh tube containing 1.5 mL of thiobarbituric acid reagent. The reaction mixture was then heated at 90 °C for 20 min. Absorbance was recorded at three wavelengths (450, 532, and 600 nm).

### 2.6. GUS Staining and GUS Activity

Histochemical GUS staining was performed on roots of *CYCB1_pro_:GUS Arabidopsis* seedlings [20]. Staining was conducted for 6 h at 37 °C in darkness using a GUS staining solution (Solarbio, Beijing, China). Images of stained root tips were captured with an Axio Observer 3 microscope (Carl Zeiss).

GUS activity was quantified by the fluorogenic substrate 4-methylumbelliferyl-β-D-glucuronide [20]. Fluorescence emission was recorded at 455 nm (excitation, 365 nm) on a BioTek Cytation 3 microplate reader (Winooski, VT, USA) after a 30 min incubation. Enzyme activity was normalized to untreated controls and expressed as relative fluorescence units.

### 2.7. Antioxidant Enzyme Activity Assay

The activities of catalase (CAT), ascorbate peroxidase (APX), and superoxide dismutase (SOD) were assayed following previously described protocols [25]. The amount of enzyme corresponding to 50% inhibition of NBT photoreduction was defined as one unit (U) of SOD activity. All enzyme activities were normalized to the soluble protein content of the samples, which was determined with a BCA Protein Assay Kit (Vazyme, Nanjing, China).

### 2.8. RT-qPCR Analysis

RNA was extracted from *Arabidopsis* seedlings using Trizol reagent as previously described [16]. The extracted RNA was reverse transcribed into cDNA using a cDNA synthesis kit (HiScript III 1st Strand cDNA Synthesis Kit, Vazyme Biotech Co., Ltd., Nanjing, China). Reverse transcription quantitative PCR (RT-qPCR) was carried out on a Mastercycler^®^ ep realplex system (Eppendorf, Hamburg, Germany). The 2^−ΔΔCT^ method was applied for calculation, with normalization performed using *Actin2* and *UBQ10* as reference genes. The corresponding primers are provided in the Table S2.

### 2.9. Statistical Analysis

All data are expressed as mean ± standard deviation (SD). Statistical significance was determined by Student’s *t*-test or Tukey’s multiple range test (Origin 2026). The replicate number (*n*) for each experiment is specified in the corresponding figure legend.

## 3. Results

### 3.1. NAR1/H_2_ Sustains Primary Root Growth upon ABA

To evaluate the role of NAR1-derived H_2_, wild-type (WT), RNA interference lines (*RNAi-NAR1-1/2*), and 35S-driven overexpression lines (*OE-NAR1-1/2*) were employed. Similar to the previous reports [17], endogenous H_2_ levels were significantly reduced in *RNAi-NAR1-1/2* seedlings, but markedly elevated in *OE-NAR1-1/2* seedlings (Figure S3). These results clearly indicate that NAR1 positively regulates *Arabidopsis* endogenous H_2_ production.

Under normal growth conditions, primary root length was increased in *OE-NAR1* lines, and inhibited in *RNAi-NAR1* lines, when compared to those in the WT (Figure 1A–C), suggesting that NAR1/H_2_ is required for root development. In contrast, ABA exerts an opposite effect on primary root growth. When ABA was applied, root growth was simultaneously inhibited across all genotypes, but the extent of inhibition differed with *NAR1* expression levels (Figure 1A–C). Specifically, *RNAi-NAR1-1/2* seedlings showed greater reduction (~40.6% and ~31.1%) than the WT (~23.2%), whereas *OE-NAR1-1/2* seedlings showed smaller decreases (~12.6% and ~11.0%). A similar pattern was observed in altered seedling fresh weight upon ABA administration (Figure 1D). Moreover, the obvious growth inhibition observed in ABA-treated *RNAi-NAR1* lines was partially rescued by H_2_ fumigation (Figure S4), further reflecting the specific role of endogenous H_2_ in root growth regulation. Importantly, both independent lines of each genotype exhibited comparable phenotypes.

### 3.2. NAR1/H_2_ Antagonistically Regulates ABA-Inhibited Root Meristem Activity

Because primary root growth relies on cell division activity within the meristematic zone, we examined the parameters of root meristem. Under the normal growth conditions, compared to the WT, a significantly increased meristem length (Figure 2A,B), higher cell numbers (Figure 2C) and cell production rate (Figure 2D) in *OE-NAR1* seedlings was observed, whereas contrasting results appeared in *RNAi-NAR1* seedlings. Upon ABA treatment, although meristematic zones were decreased in all genotypes, the reduction was less pronounced in *OE-NAR1* seedlings, but more impaired in *RNAi-NAR1* seedlings, with consistent results across both independent lines of each genotype. These findings suggest that the NAR1-mediated changes in primary root length are attributable, at least in part, to alterations in meristem activity. In addition, for altered root cap length, we only observed this significant difference occurring between WT and *OE-NAR1-2* under ABA treatment (Figure S5).

The precise regulation of cell-cycle progression is core to maintaining meristem activity, with the G2/M transition being particularly critical for cell proliferation in root tips [10,26]. Consistent with the meristematic phenotypes, *CYCB1;1* expression (Figure 3A) and GUS activity (Figure 3B), as assessed by the *CYCB1_pro_:GUS* reporter, were altered accordingly. Although *CYCB1;1* expression was higher in *OE-NAR1* seedling and lower in *RNAi-NAR1* plants under the normal growth conditions, ABA addition reduced their levels in all genotypes, particularly showing the obvious reduction being attenuated in *OE-NAR1* plants, but exacerbated in *RNAi-NAR1* seedlings.

Notably, we also observed that H_2_ fumigation partially reversed the meristem defects observed in *RNAi-NAR1* seedlings (Figure 2 and Figure 3). These observations clearly reflected the notion that above NAR1 responses were mainly mediated via H_2_, although a possible involvement of cytosolic Fe-S cluster assembly achieved by NAR1 could not be easily ruled out.

### 3.3. NAR1/H_2_ Alleviates ABA-Induced Oxidative Stress

ROS are critical for regulating cell division in the root meristem. To determine whether NAR1 acts through regulation of redox homeostasis, H_2_O_2_ and O_2_^−^ levels were monitored by DAB and NBT staining, respectively. Under the normal growth conditions, no differences in ROS accumulation were apparently observed among different genotypes (Figure 4A,B). Upon ABA treatment, however, staining intensity of DAB and NBT staining showed different patterns. For example, compared with WT, *OE-NAR1* lines exhibited lighter staining, whereas *RNAi-NAR1* lines showed deeper coloration.

Levels of ROS in root tissues were further quantified by using the fluorescent indicator H_2_DCFDA, yielding the similar results comparable to the above two staining patterns (Figure 4C,D). In addition, ABA-induced lipid peroxidation, measured by TBARS content, was attenuated in *OE-NAR1* lines but exacerbated in *RNAi-NAR1* lines (Figure 4E). More importantly, we clearly observed that the H_2_ fumigation partially reversed the oxidative damage in *RNAi-NAR1* seedlings, establishing that NAR1-catalyzed endogenous H_2_ might be an important contributor to the maintenance of cellular redox homeostasis upon ABA stress.

Superoxide dismutase (SOD) catalyzes the dismutation of O_2_^−^ into O_2_ and H_2_O_2_, with the latter being subsequently reduced to water through the action of catalase (CAT) and ascorbate peroxidase (APX) [27]. To explore the molecular mechanism, we determined the expression and enzymatic activities of key ROS-scavenging enzymes. First, the transcript levels of *CSD1*/*CSD3* (encoding Cu/Zn SOD), *CAT3* (encoding CAT), and *APX1*/*APX2* (encoding APX) were examined. Similar to the altered ROS accumulation patterns under ABA treatment, the transcript levels of above ROS scavenging genes were differentially upregulated in *OE-NAR1* lines where endogenous H_2_ levels are enhanced (Figure 5A). The knockdown of *NAR1* in RNAi lines, including *RNAi-NAR1-1* and *RNAi-NAR1-2*, simultaneously impaired the expression of these genes. Importantly, application of H_2_ fumigation partially rescued the expression level in *RNAi-NAR1* lines. Since the enzymatic activities of SOD, CAT, and APX showed similar tendencies (Figure 5B–D), the above results clearly established that NAR1-mediated regulation of ROS-scavenging genes is partly dependent on endogenous H_2_. Taken together, these results led us to conclude that NAR1-mediated H_2_ production enhances ROS-scavenging gene expression and reduces ROS accumulation, thereby enabling NAR1 to reshape ABA-induced growth inhibition.

### 3.4. Auxin Signaling Is Altered by NAR1/H_2_ upon ABA

Given that auxin signaling is critical for root cell division and growth and is inhibited by ABA, we examined whether NAR1 modulates ABA sensitivity through this pathway. To monitor auxin response, the *DR5rev:3xVENUS-N7* reporter system was employed. Confocal imaging analysis showed that DR5 fluorescence intensity was clearly increased in *OE-NAR1* seedling and decreased in *RNAi-NAR1* compared to the WT under normal growth conditions (Figure 6A,B), matching the observed root length phenotypes (Figure 1A). Upon ABA treatment, compared to the normal growth condition, DR5 intensity was obviously reduced across all genotypes. However, the most pronounced reduction was observed in the *RNAi-NAR1* line, whereas the *OE-NAR1* line exhibited a less severe decrease. In agreement with corresponding phenotypes (Figure S4), the suppressed DR5 signal in *RNAi-NAR1* was partially restored by H_2_ fumigation.

It is well documented that auxin transport is mediated by influx and efflux carriers. Specifically, the PIN protein family facilitates auxin efflux, while AUX1 acts as an influx carrier to mediate auxin entry into cells. To determine whether the changes in auxin signaling were accompanied by alterations in auxin transport, we further examined the fluorescence of the auxin carriers AUX1, PIN1, and PIN2. In the absence of ABA, as anticipated, a marked enhancement of AUX1 fluorescence was observed in the *OE-NAR1* background, whereas *RNAi-NAR1* line displayed a weaker signal than the WT (Figure 7A,B). When challenged with ABA, AUX1 intensity declined across all genotypes, with the reduction being most pronounced in *RNAi-NAR1* line and least in *OE-NAR1* line, thereby further amplifying the differences between them. Notably, the suppressed AUX1 signal in *RNAi-NAR1* was partially rescued by H_2_ fumigation. Similar patterns were observed for PIN1 (Figure 7C,D) and PIN2 (Figure 7E,F). Together, the NAR1-dependent regulation of these carriers was evident under both control and ABA-treated conditions, clearly establishing that NAR1 modulates auxin transport by influencing the abundance of both influx and efflux carriers. Hence, these alterations in auxin transport, together with the changes in auxin signaling, may contribute to the differential ABA sensitivity observed among different NAR1 genetic lines.

Collectively, our findings reveal that NAR1-catalyzed endogenous H_2_ production enhances root plasticity under ABA stress (Figure 8). This effect is mainly attributed to elevated meristem cell division activity, together with concomitant alterations in auxin signaling and ROS homeostasis, thus facilitating root adaptive growth.

## 4. Discussion

The capacity of root systems to reshape their architecture in response to fluctuating soil nutrient landscapes underpins plant fitness and resource acquisition [1]. Here, using *Arabidopsis* hydrogenase NAR1-related genetic lines [17], including *RNAi-NAR1-1/2* and *OE-NAR1-1/2* lines, we found that NAR1 positively reshapes root growth, with corresponding changes in RAM length and cell number, particularly upon ABA stress (Figure 1 and Figure 2). Under ABA treatment, root growth inhibition is closely associated with reduced RAM cell division activity, partly due to impaired G2/M transition [10,26,28]. Notably, NAR1 modulates the expression of *CYCB1;1*, a G2/M marker gene, suggesting that NAR1 attenuated ABA-induced cell-cycle suppression (Figure 3). Although H_2_ has been reported to promote the transcription of cell-cycle genes, these findings were largely based on exogenous H_2_ application or heterologous expression systems [15,16,29]. Here, NAR1-related genetic lines were employed in this study, providing more direct genetic evidence for the role of biogenic H_2_ in root development. Our results clearly show that, besides its known roles in floral transition and lateral root branching, NAR1 definitely contributes to sustaining root meristem cell division activity when growth is challenged by ABA, a stress-induced phytohormone [30].

The auxin-signaling network is intimately connected to cell-division activity in the RAM [1,2,31]. Previous studies have indicated that auxin accumulation and transport in root tips are inhibited by ABA, leading to the suppression of meristematic cell division [6,7,10]. In this study, enhanced auxin signaling (indicated by DR5 reporter fluorescence) was observed in *OE-NAR1* line under normal conditions. Relatively high signal levels were maintained in *OE-NAR1* line even under ABA treatment, whereas a significant decrease in auxin signaling was detected in *RNAi-NAR1* line regardless of ABA addition (Figure 6). In line with previous findings [28,32,33], the expression of auxin transporters was inhibited by ABA (Figure 7). We further found that the expression of AUX1, PIN1, and PIN2 is positively regulated by NAR1. Similarly, in studies of tomato lateral root development, local auxin accumulation was found to be promoted by NAR1 through the regulation of AUX1 and PINs expression [18]. These results were consistent with the observed alterations in root length and RAM phenotypes (Figure 1 and Figure 2), suggesting that the auxin-signaling network is efficiently maintained by NAR1-driven H_2_ in response to ABA addition or environmental stresses.

Excess ROS can inhibit cell proliferation by interfering with DNA replication and affecting auxin transport [34,35,36]. In *Arabidopsis* root tips, ABA treatment has been shown to induce H_2_O_2_ and O_2_^−^ accumulation [28]. Here, *OE-NAR1* lines exhibited relatively lower ROS and lipid peroxidation levels under ABA treatment, whereas *RNAi-NAR1* lines showed the opposite trend (Figure 4). In plants, the scavenging of ROS can be achieved through enzymatic antioxidant defense pathways [27,37]. Altered expression and enzymatic activities of SOD, CAT, and APX were clearly observed in *NAR1* lines (Figure 5), correlating with the accompanying changes in ROS levels. These results clearly suggested that NAR1 enhances ROS-scavenging capacity under ABA stress.

Notably, H_2_ fumigation partially rescued the oxidative damage in *RNAi-NAR1* lines (Figure 4), a finding that parallels the selective antioxidant effects of H_2_ described in the medical field [38,39] and later confirmed in plants [40,41]. Together with these previous reports, our observations suggest a possible role for endogenous H_2_ as a signaling molecule linking redox homeostasis to root developmental plasticity under stress. Indeed, no direct interactions between *Arabidopsis* and tomato NAR1 and auxin transport-related proteins or related transcription factors were observed in our yeast two-hybrid screening [18]. Besides the direct effect of ROS on cell-cycle progression, it also affects cell division activity through alteration of auxin homeostasis, particularly under stress conditions such as ABA treatment [7,36,42]. Therefore, the causal relationship between ROS and auxin signaling and the direct or indirect contribution in our experimental system remains to be further investigated.

Given that NAR1 plays a dual role in H_2_ production and Fe-S cluster assembly [17,18,43], complementation experiments were performed by applying H_2_ fumigation. This treatment partially rescued the root growth defects in *RNAi-NAR1* lines (Figure 2 and Figure S4). Previous studies found that heterologous expression of *CrHYD1* in *Arabidopsis*, not only elevates endogenous H_2_ production, but also promotes root development and modulates auxin signaling [15,16]. Recent evidence clearly showed that the *mms19* mutant, defective in Fe-S cluster assembly, exhibits early flowering regardless of H_2_ supplementation, whereas the late-flowering phenotype of *nar1* mutants is effectively rescued by exogenous H_2_ [17]. A similar exogenous H_2_ rescue effect has been confirmed in tomato *nar1* mutants with respect to lateral root branching [18]. These data support a specific role of endogenous H_2_ in the observed phenotypes, which were partially independent of Fe-S-related effects. Since H_2_ specifically targets [2Fe-2S] cluster of Rieske iron-sulfur protein in mice, promoting its degradation, while other Fe-S proteins appear unaffected [44], whether or how the positive effects of *Arabidopsis* NAR1-dependent H_2_ are also functionally associated with the altered redox/Fe-S status remains an open question.

## 5. Conclusions

In summary, our findings indicate that NAR1-catalyzed endogenous H_2_ enhances root plasticity by promoting meristem cell division activity, as well as by modulating auxin signaling and ROS homeostasis under ABA-challenged conditions. These observations provide new insights into the physiological functions of biogenic H_2_ in plants and possible application in crops under a variety of agricultural conditions.

## Figures and Tables

**Figure 1:**
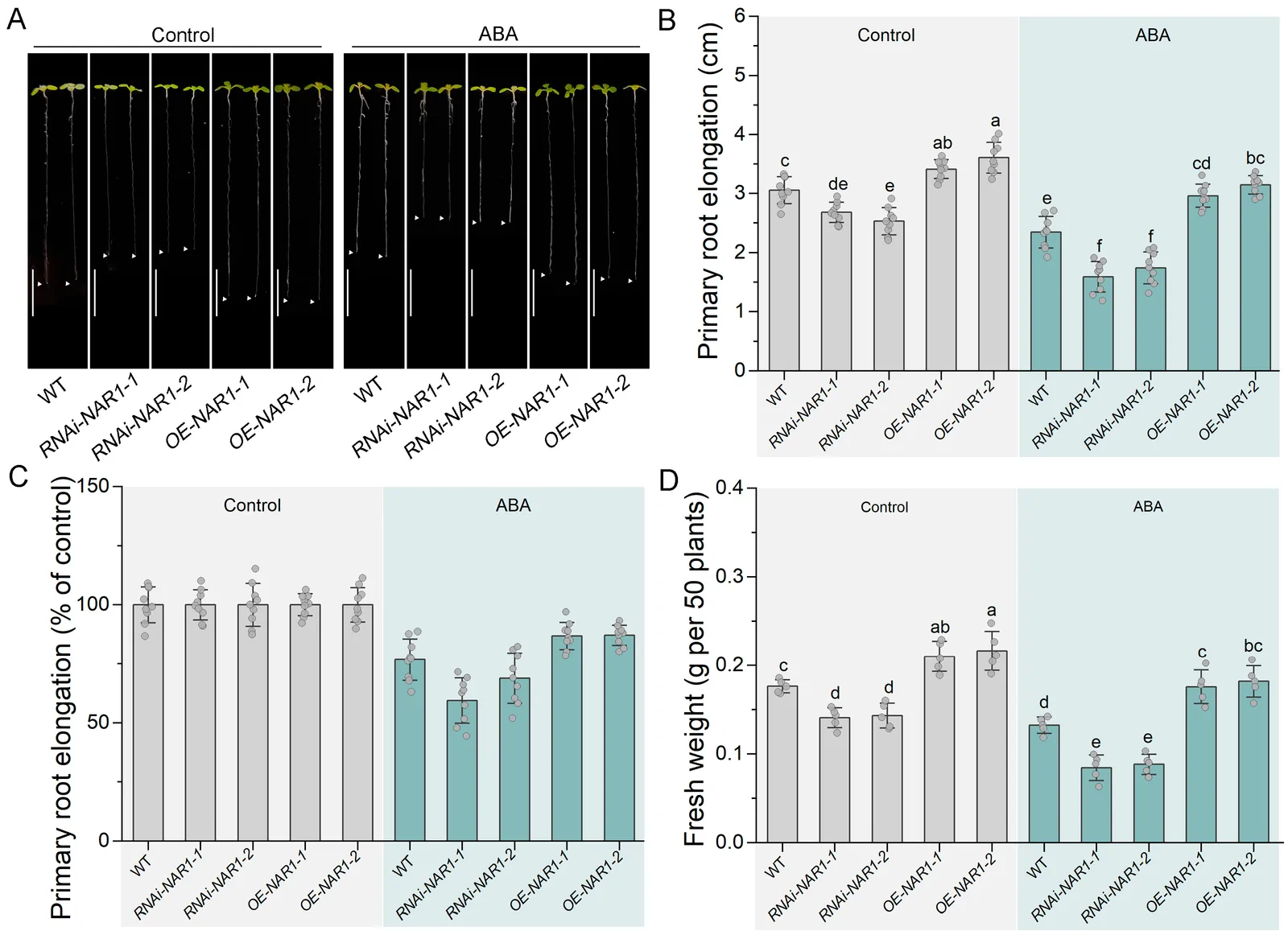
ABA-impaired root plasticity was altered by *NAR1*. Three-day-old *Arabidopsis* seedlings of wild-type (WT), *RNAi-NAR1-1/2*, and *OE-NAR1-1/2* were transferred to ½MS solid medium without (Control) or with ABA for another 7 days. (**A**) Representative images of seedling phenotypes. Scale bar, 1 cm. The arrowheads indicate the root tips. (**B**) Primary root elongation during the 7-day treatment (*n* = 9), and (**C**) was normalized to growth under the corresponding control conditions (set as 100%; *n* = 9). (**D**) Seedling fresh weight per 50 plants (*n* = 5). Data are presented as mean ± SD. Different letters denote statistical significance (*p* < 0.05) by Tukey’s multiple range test.

**Figure 2:**
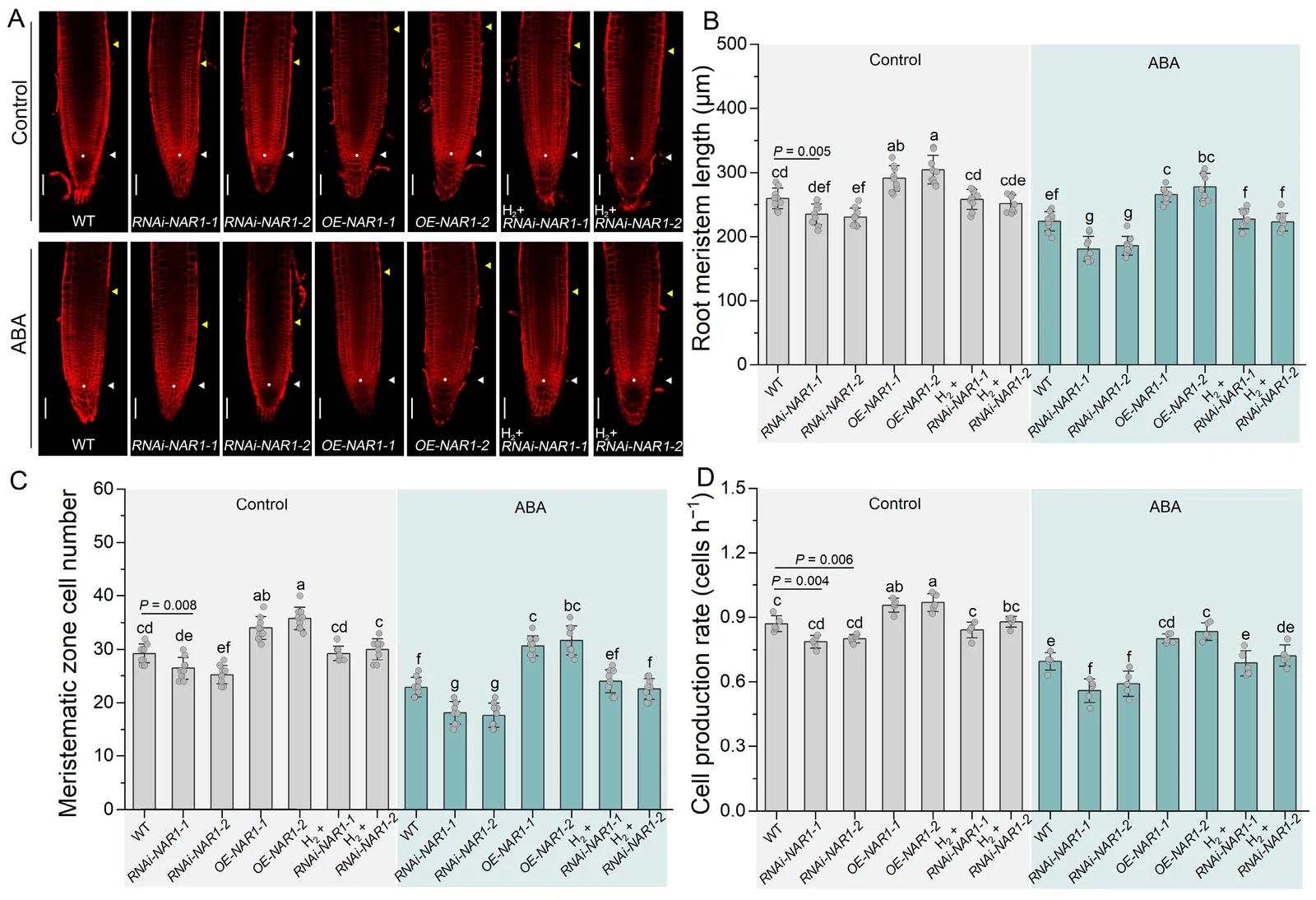
Alteration of ABA-inhibited response in root meristematic zone by *NAR1*. Three-day-old seedlings were treated without (Control) or with ABA, or 0.5% (*v*/*v*) H_2_ fumigation, either alone or in combination, and grown for another 7 days. (**A**) Representative FM4-64-stained images of root meristem. White triangles indicate the quiescent center (QC), and white dots are placed on the QC region for marking its position. Yellow triangles indicate the boundary between the meristematic and elongation zones. The meristematic zone was defined as the region from the QC to the first rectangular cell in the cortex. Scale bar, 50 µm. (**B**) Root meristem length (*n* = 9). (**C**) Number of cells in the meristem zone (*n* = 9). (**D**) Cell production rate (*n* = 5). Data are presented as mean ± SD. Different letters denote statistical significance (*p* < 0.05) by Tukey’s multiple range test. Statistical significance was also determined using Student’s *t*-test, and *p*-values were calculated.

**Figure 3:**
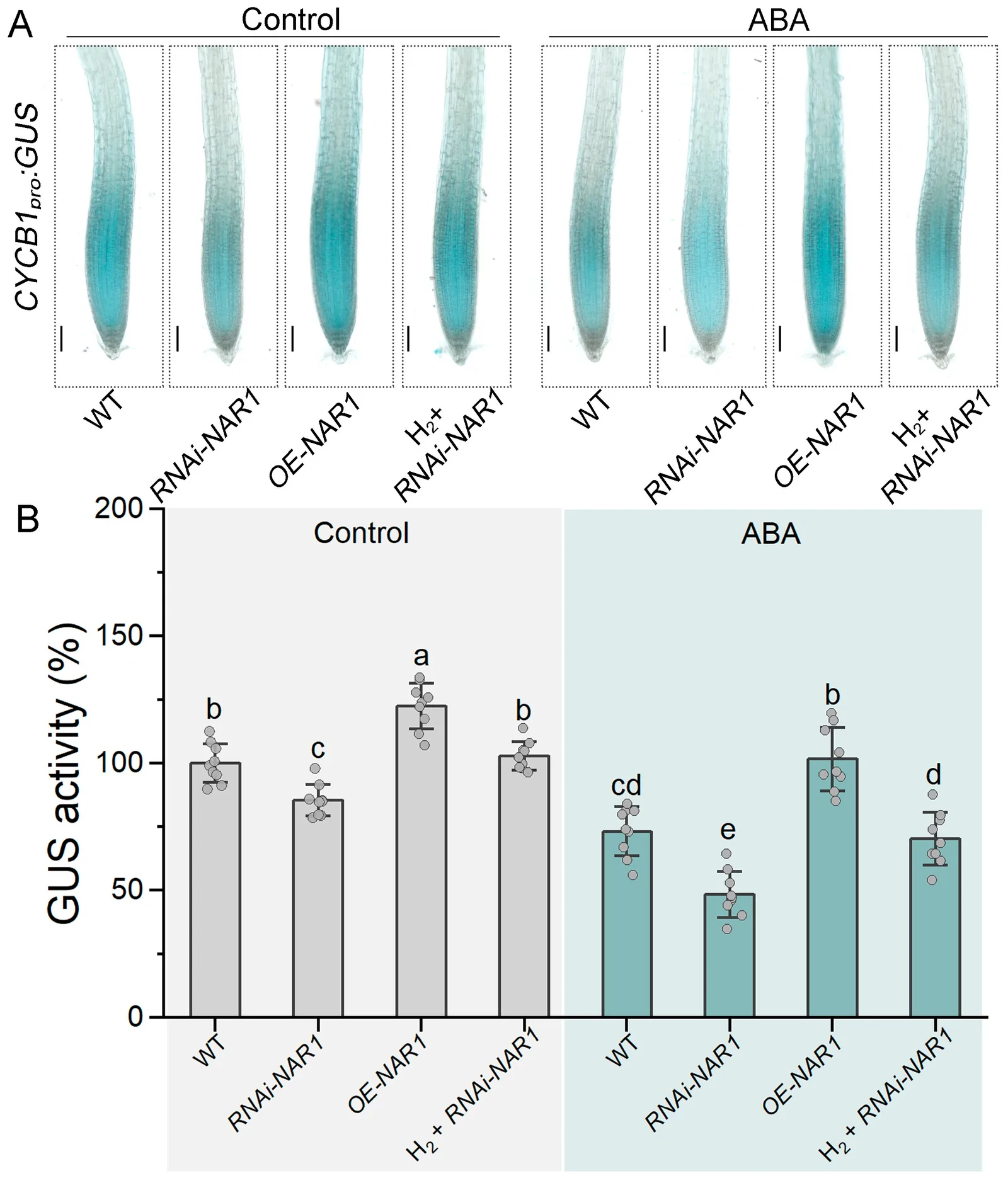
ABA-impaired root cell division is rescued by NAR1-catalyzed H_2_. Three-day-old seedlings were treated without (Control) or with ABA, or 0.5% (*v*/*v*) H_2_ fumigation, either alone or in combination, and grown for another 3 days. (**A**) *CYCB1_pro_:GUS* expression in the root tip. Scale bar, 100 μm. (**B**) GUS activity (*n* = 9). Data are presented as mean ± SD. Different letters denote statistical significance (*p* < 0.05) by Tukey’s multiple range test.

**Figure 4:**
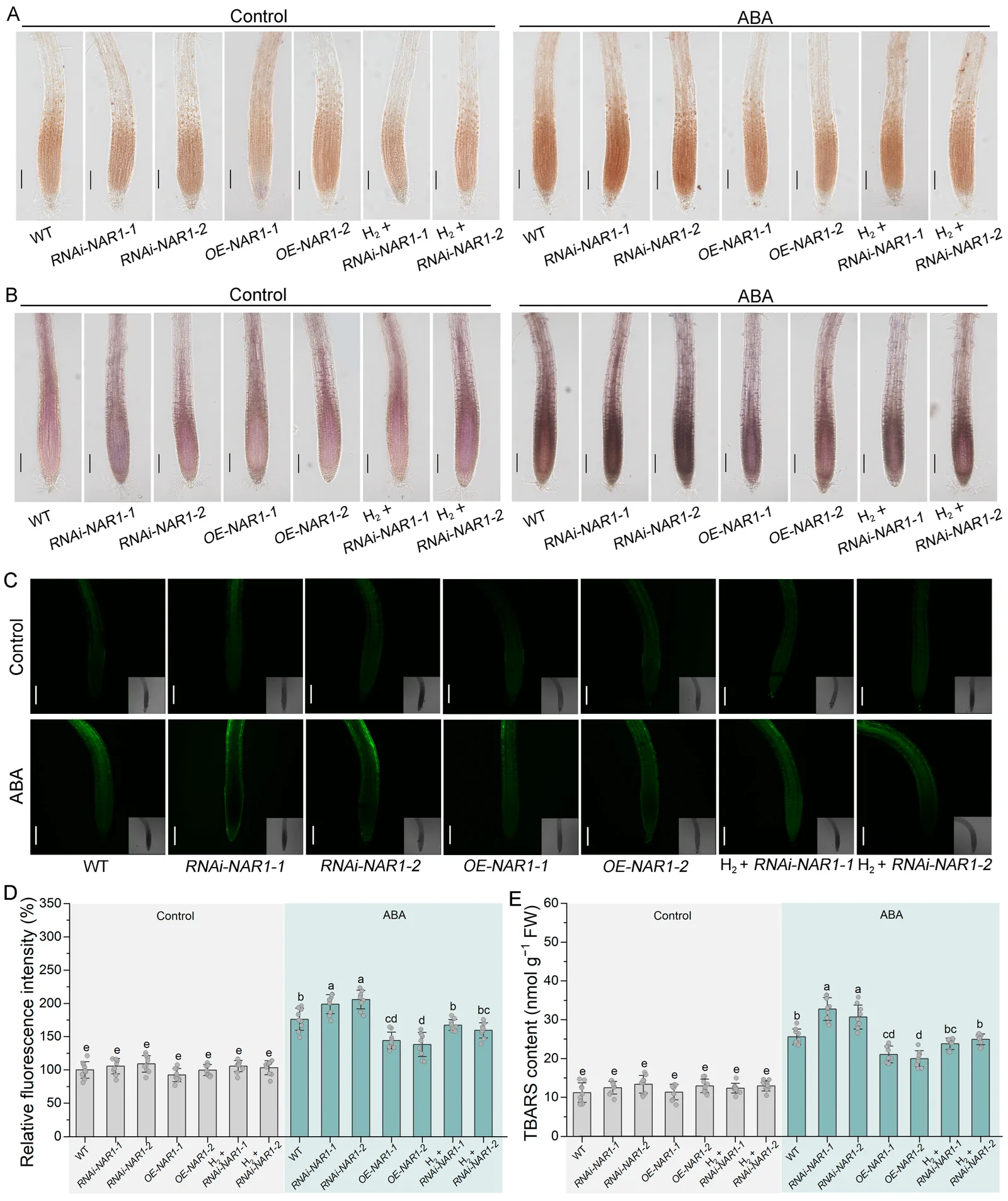
ABA-induced root oxidative damage is alleviated by NAR1-mediated H_2_. Three-day-old seedlings were treated without (Control) or with ABA, or 0.5% (*v*/*v*) H_2_ fumigation, either alone or in combination, and grown for another 3 days. (**A**) DAB staining. Scale bar, 200 μm. (**B**) NBT staining. Scale bar, 200 μm. (**C**) Representative images of H_2_DCFDA fluorescence in roots. Scale bar, 100 μm. (**D**) Relative fluorescence intensity (*n* = 9). (**E**) TBARS content (*n* = 9). Data are presented as mean ± SD. Different letters denote statistical significance (*p* < 0.05) by Tukey’s multiple range test.

**Figure 5:**
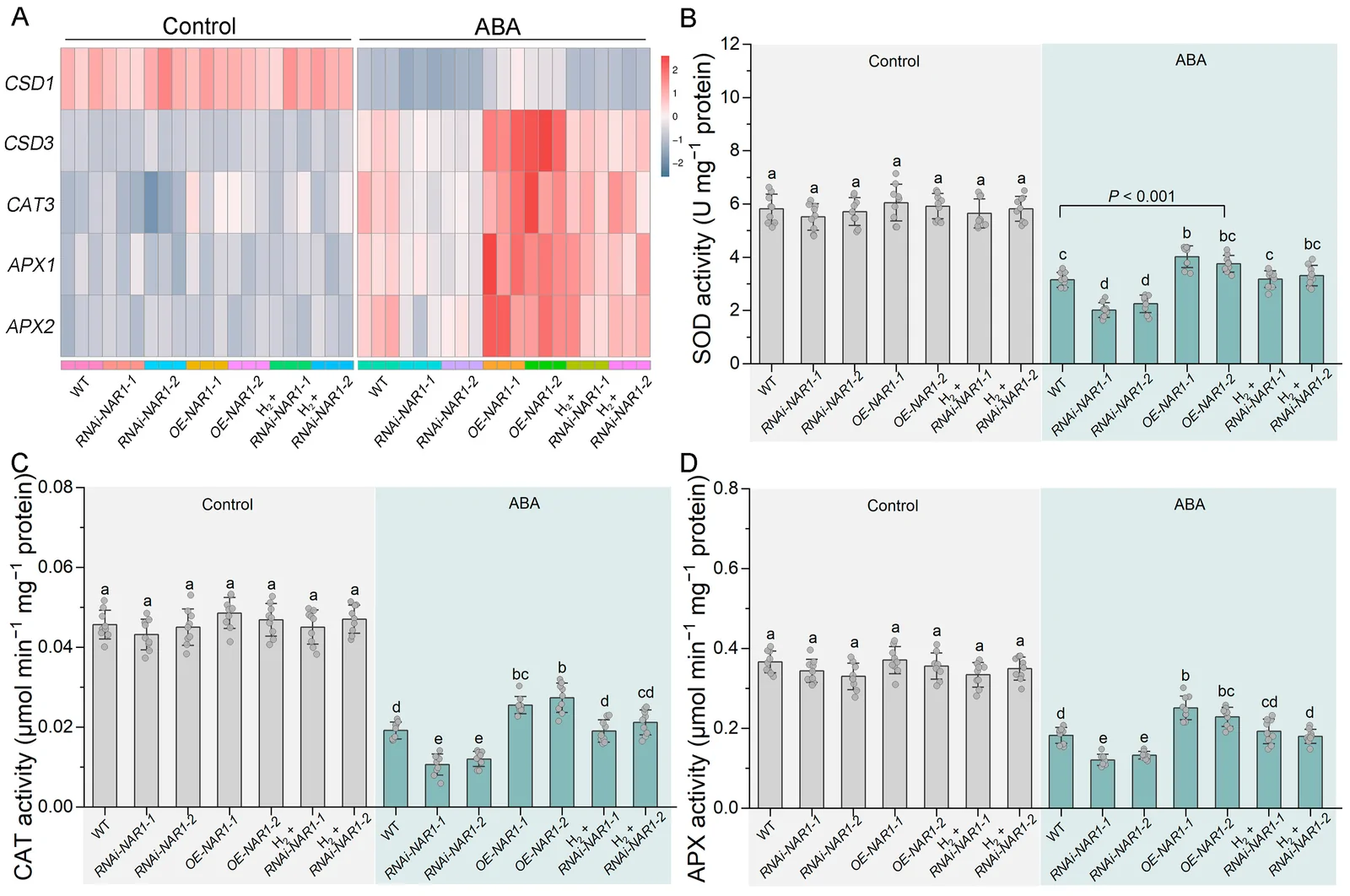
Altered ROS scavenging capacity by NAR1. Three-day-old seedlings were treated without (Control) or with ABA, or 0.5% (*v*/*v*) H_2_ fumigation, either alone or in combination, and grown for another 3 days. (**A**) The relative expression levels of ROS scavenging-related genes, including *CSD1*, *CSD3*, *CAT3*, *APX1*, and *APX2*, were determined (*n* = 3). (**B**–**D**) The activities of SOD, CAT, and APX were analyzed (*n* = 9). Data are presented as mean ± SD. Different letters denote statistical significance (*p* < 0.05) by Tukey’s multiple range test. Statistical significance was also determined using Student’s *t*-test, and *p*-values were calculated.

**Figure 6:**
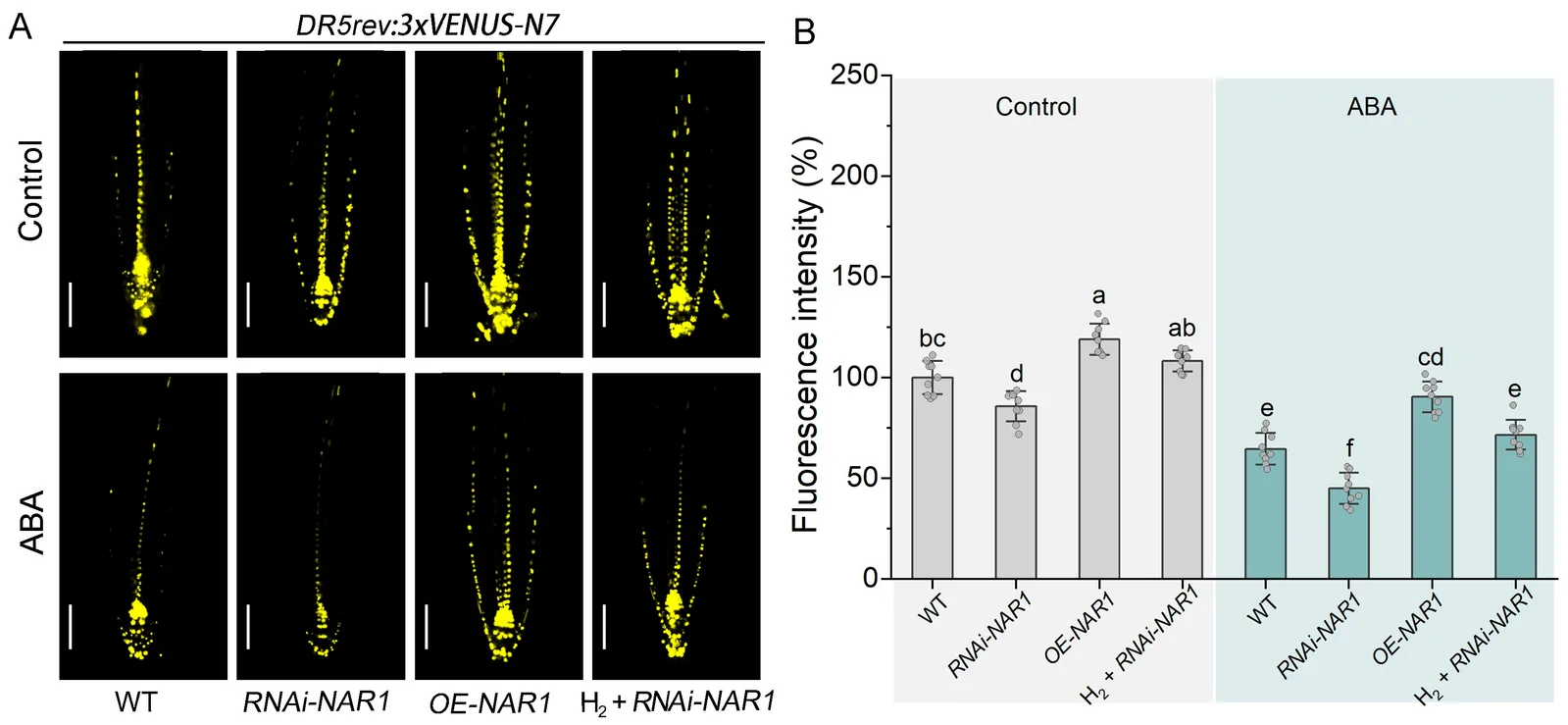
Auxin response is altered by NAR1. Three-day-old seedlings were treated without (Control) or with ABA, or 0.5% (*v*/*v*) H_2_ fumigation, either alone or in combination, and grown for another 3 days. (**A**) Confocal images of *DR5rev:3xVENUS-N7* in roots. Scale bar, 50 μm. (**B**) Relative fluorescence intensity (*n* = 9). Data are presented as mean ± SD. Different letters denote statistical significance (*p* < 0.05) by Tukey’s multiple range test.

**Figure 7:**
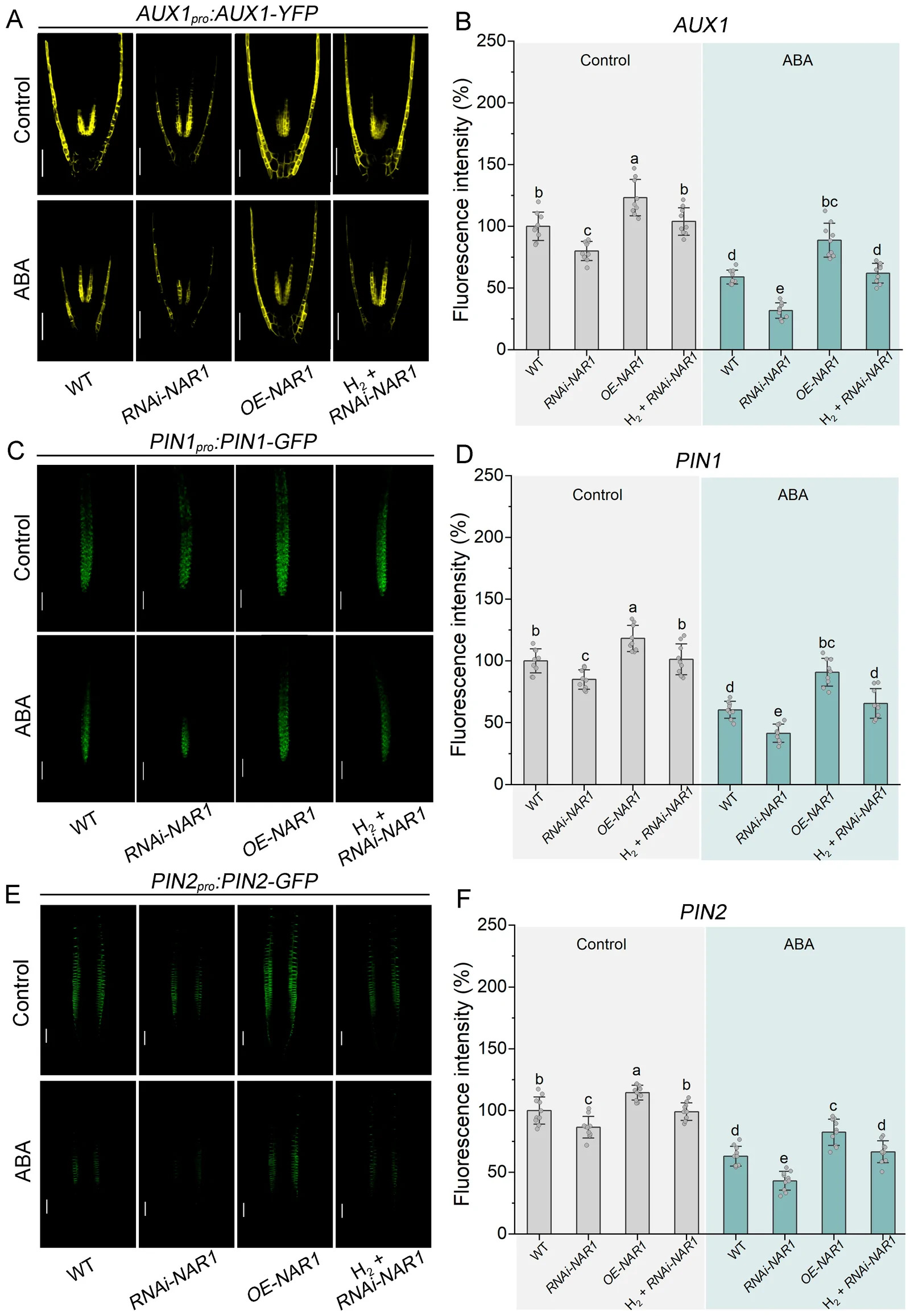
Regulation of auxin transport by NAR1-mediated H_2_. Three-day-old seedlings were treated without (Control) or with ABA, or 0.5% (*v*/*v*) H_2_ fumigation, either alone or in combination, and grown for another 3 days. (**A**–**F**) Confocal images (**A**,**C**,**E**) and relative fluorescence intensity (**B**,**D**,**F**) of *AUX1_pro_:AUX1-YFP*, *PIN1_pro_:PIN1-GFP*, and *PIN2_pro_:PIN2-GFP* in roots (*n* = 9). Scale bar, 50 μm. Data are presented as mean ± SD. Different letters denote statistical significance (*p* < 0.05) by Tukey’s multiple range test.

**Figure 8:**
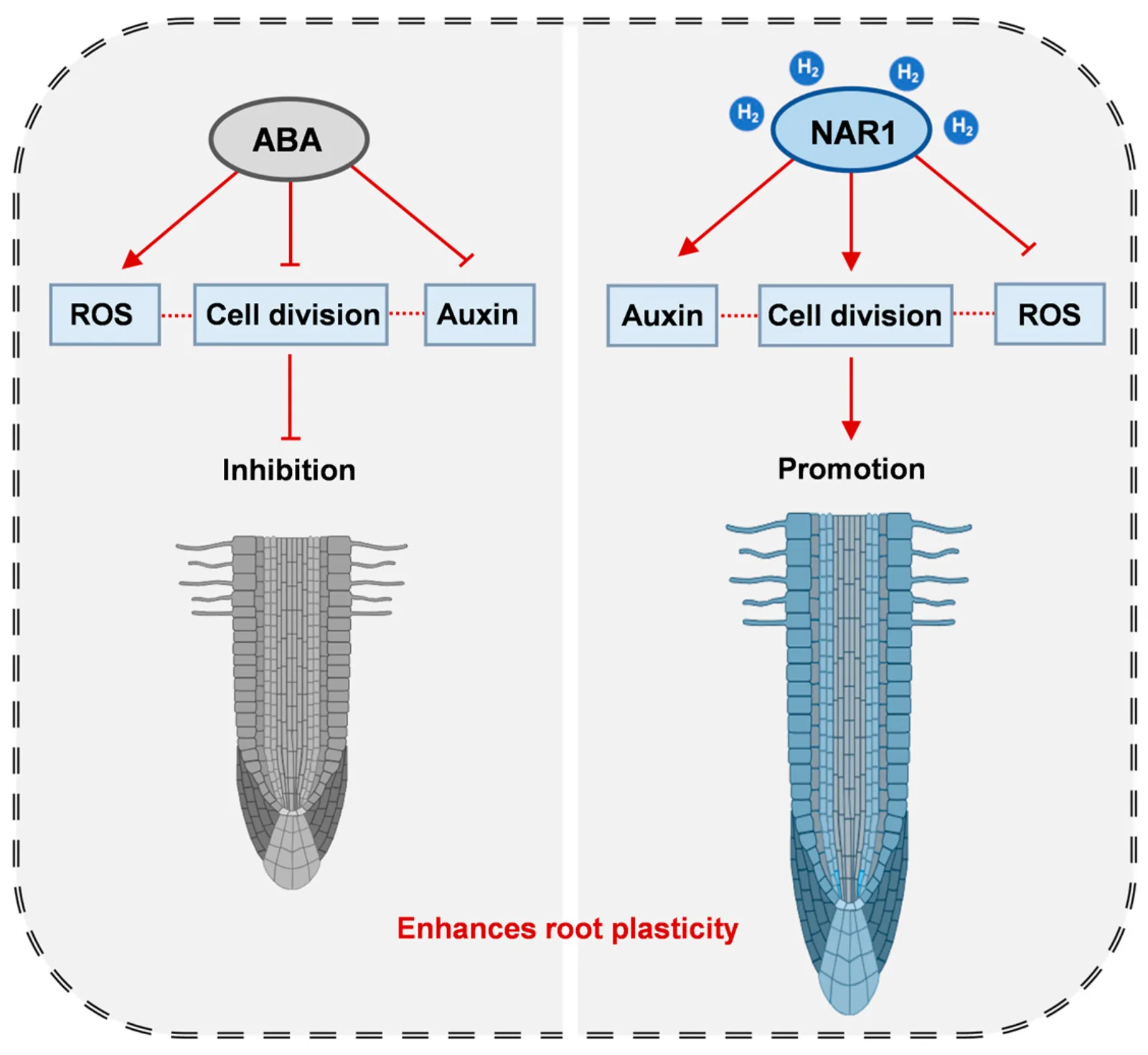
A working model of NAR1-catalyzed H_2_ in reshaping root plasticity under ABA stress. NAR1 enhances root meristem cell division activity, accompanied by altered auxin signaling and ROS homeostasis, both of which collectively promote root adaptive growth. Arrow indicates positive regulation, T-bar denotes inhibition, and the dashed line represents a previously reported connection. Partial elements of this figure were created by Figdraw (www.figdraw.com, ID:ORROI6e72e, accessed on 6 April 2026).

## Data Availability

The original contributions presented in this study are included in the article/Supplementary Material. Further inquiries can be directed to the corresponding author.

## Supplementary Materials

The following supporting information can be downloaded at: https://www.mdpi.com/article/10.3390/antiox15101228/s1, Table S1: PCR primers for plant material identification; Table S2: Primer sequence for RT-Qpcr; Figure S1: Effects of different concentrations of ABA on primary root elongation; Figure S2: Effects of H_2_ fumigation on primary root elongation in *RNAi-NAR1* lines under ABA treatment; Figure S3: Altered H_2_ levels by *NAR1*; Figure S4: Mutation of NAR1-influenced and ABA-dependent primary root growth inhibition was restored by exogenous H_2_; Figure S5: Alteration of ABA-inhibited root cap length by *NAR1*.

## Abbreviations

The following abbreviations are used in this manuscript:

½MS	Half-strength Murashige and Skoog
ABA	Abscisic acid
APX	Ascorbate peroxidase
CAT	Catalase
DAB	3,3′-diaminobenzidine
H_2_	Molecular hydrogen
H_2_DCFDA	2′,7′-Dichlorodihydrofluorescein diacetate
H_2_O_2_	Hydrogen peroxide
NAR1	Nuclear Architecture Related 1
NBT	Nitro blue tetrazolium
O_2_^-^	Superoxide anion
OE	Overexpression
PIN	PIN-FORMED
QC	Quiescent center
RAM	Root apical meristem
RNAi	RNA interference
ROS	Reactive oxygen species
RSA	Root system architecture
RT-qPCR	Reverse transcription quantitative PCR
SD	Standard deviation
SOD	Superoxide dismutase
TBARS	Thiobarbituric acid reactive substances
WT	Wild-type
