# Supplementary material for: Nuclear Architecture Related 1-Dependent H2 Reshapes Root Plasticity Through Targeting Root Meristem Cell Division Activity
Source: Antioxidants (Basel). 2026 Sep 23;15(10):1228. doi: 10.3390/antiox15101228 (PMC13700628; doi:10.3390/antiox15101228)
Supplement: Supplementary file 1 [file antioxidants-15-01228-s001.zip › antioxidants-4458677-supplementary.pdf]

**Supplementary Materials**

# Nuclear Architecture Related 1-Dependent $H_2$ Reshapes Root Plasticity Through Targeting Root Meristem Cell Division Activity

**Ke Jiang [1], Pengfei Cheng [1], Ziyu Liu [1], Hongmei Du [2] and Wenbiao Shen [1,*]**

1 Laboratory Center of Life Sciences, College of Life Sciences, Nanjing Agricultural University, Nanjing 210095, China; 2022216005@stu.njau.edu.cn (K.J.); t2024051@njau.edu.cn (P.C.); 2025216044@stu.njau.edu.cn (Z.L.)

2 School of Design, Shanghai Jiao Tong University, Shanghai 200240, China; hmdu@sjtu.edu.cn

* Correspondence: wbshenh@njau.edu.cn

**Table S1.** PCR primers for plant material identification.

| Primer | Sequence |
|---|---|
| *OE-NAR1-identification-F* | ACGTAAGGGATGACGCACAA |
| *OE-NAR1-identification-R* | CCATGTATTCTCCTCCTGCCC |
| *RNAi-NAR1-identification-F* | TTCATTTGGAGAGAACACGGGGGAC |
| *RNAi-NAR1-identification-R* | GGGCTCTGTAACTATC |

**Table S2.** Primer sequence for RT-qPCR.

| Primer | Sequence |
|---|---|
| *Actin2-F* | AGTGGTCGTACAACCGGTATTGT |
| *Actin2-R* | GATGGCATGAGGAAGAGAGAAAC |
| *UBQ10-F* | GGTGGTTTGTGTTTTGGGGC |
| *UBQ10-R* | AGTCGAGTCACTTTGCAGGC |
| *CSD1-F* | TTTTGGACCTCGTGGGCTAT |
| *CSD1-R* | TGGCCTTTGTGATCTCAGGA |
| *CSD3-F* | TCATGCTGGCGATTTGGGTA |
| *CSD3-R* | GCTAAGCTTGTGCCCTCCTT |
| *CAT3-F* | TGTACCCTTCCACTTCGGGA |
| *CAT3-R* | GCGCTTGAAGGACGATACTTG |
| *APX1-F* | GCACTATTGGACGACCCTGT |
| *APX1-R* | GCATCAGCAAACCCAAGCTC |
| *APX2-F* | TAAAGCCTTCAACGATTTCCAGA |
| *APX2-R* | CAGCCGAGTGCCATGCAA |

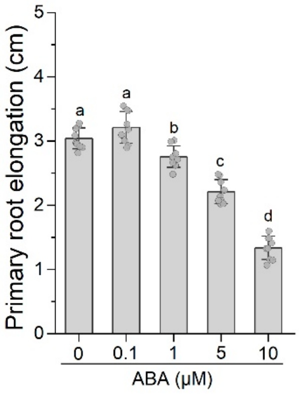


**Figure S1.** Effects of different concentrations of ABA on primary root elongation. Three-day-old *Arabidopsis* seedlings of wild-type (WT) were transferred to ½MS solid medium with indicated concentrations of ABA (0, 0.1, 1, 5, and 10 μM) for another 7 days. Primary root elongation during the 7-day treatment was measured (n = 9). Data are presented as mean ± SD. Different letters denote statistical significance ($P < 0.05$) by Tukey's multiple range test.

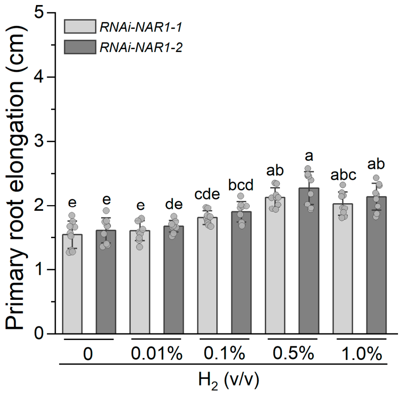


**Figure S2.** Effects of $H_2$ fumigation on primary root elongation in *RNAi-NAR1* lines under ABA treatment. Three-day-old *RNAi-NAR1-1* and *RNAi-NAR1-2* seedlings were transferred to ½MS solid medium containing 5 μM ABA and fumigated with 0, 0.01%, 0.1%, 0.5%, or 1.0% $H_2$ (v/v) for another 7 days. Primary root elongation during the 7-day treatment was measured (n = 9). Data are presented as mean ± SD. Different letters denote statistical significance ($P < 0.05$) by Tukey's multiple range test.

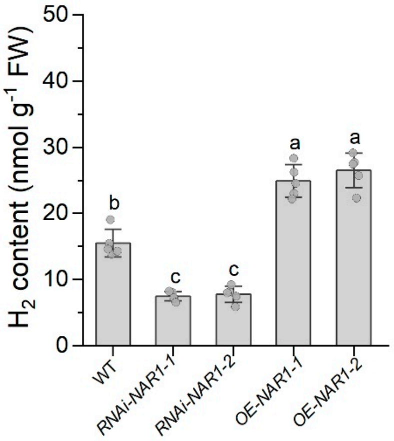


**Figure S3.** Altered $H_2$ levels by *NAR1*. $H_2$ levels were detected in ten-day-old *Arabidopsis* seedlings of WT, *RNAi-NAR1-1/2*, and *OE-NAR1-1/2* (n = 5). Data are presented as mean ± SD. Different letters denote statistical significance ($P < 0.05$) by Tukey's multiple range test.

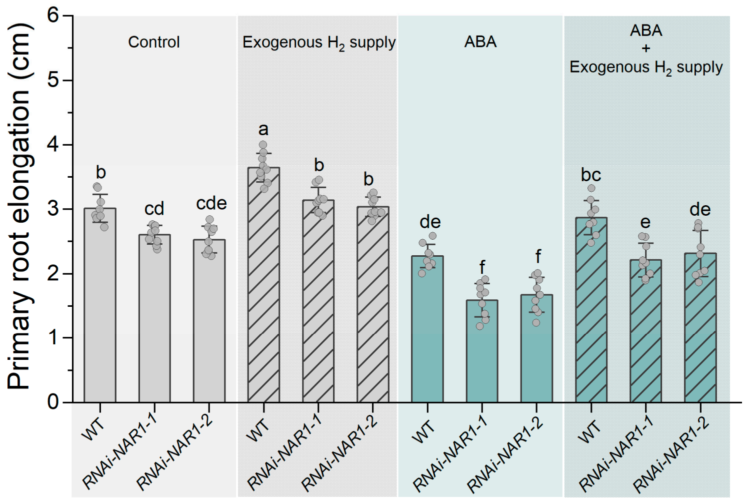


**Figure S4.** Mutation of NAR1-influenced and ABA-dependent primary root growth inhibition was restored by exogenous $H_2$. Three-day-old WT and *RNAi-NAR1-1/2* seedlings were transferred to ½MS solid medium without (Control) or with ABA, or 0.5% (v/v) $H_2$ fumigation, alone or in combination. Primary root elongation was measured over a 7-day treatment period (n = 9). Data are presented as mean ± SD. Different letters denote statistical significance ($P < 0.05$) by Tukey's multiple range test.

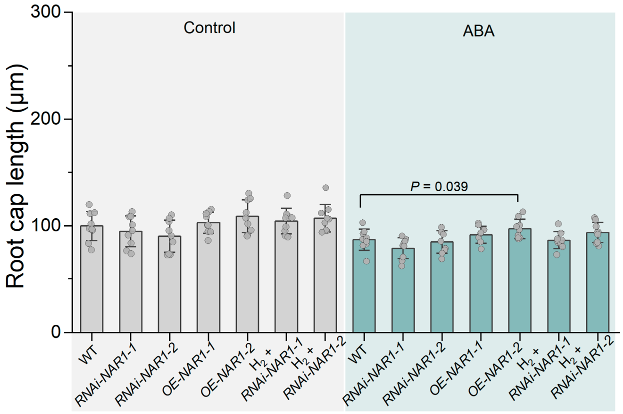


**Figure S5.** Alteration of ABA-inhibited root cap length by *NAR1*. Three-day-old seedlings were treated without (Control) or with ABA, or 0.5% (v/v) $H_2$ fumigation, either alone or in combination, and grown for another 7 days. The length of the root cap was measured from the QC to the last intact layer (separating layer was not included) (n = 9). Data are presented as mean ± SD. Statistical significance was determined using Student's *t*-test, and *P*-values were calculated.
